# Percutaneous resection of metastatic renal cell carcinoma

**DOI:** 10.1590/S1677-5538.IBJU.2018.0490

**Published:** 2019-07-27

**Authors:** Amir Toussi, Deepak Agarwal, Bradley Leibovich, Aaron Potretzke

**Affiliations:** 1 Department of Urology, Mayo Clinic, Rochester, MN, United States

## Abstract

**Introduction:**

Metastasis-directed therapy of small solitary foci of metastatic renal cell carcinoma has been associated with improved survival. Percutaneous resection of tumors in the upper tract urinary system has been widely used for treatment of localized urothelial carcinoma, however, its role in renal cell carcinoma has not been described. Herein, we present the first case of patient undergoing percutaneous resection of renal cell carcinoma in the contralateral renal pelvis.

**Materials and Methods:**

This is a case report describing the diagnosis, management and surgical approach to renal cell carcinoma recurrence in the contralateral renal pelvis.

**Results:**

Our patient was a 75-year-old male with a history of renal cell carcinoma status post radical nephrectomy who developed a solitary 2 cm recurrence in the contralateral renal pelvis, which was found after he presented with gross hematuria. He underwent successful percutaneous resection of this recurrence with final pathology showing clear cell renal cell carcinoma.

**Conclusion:**

We present the first case of renal cell carcinoma recurrence in the contralateral renal pelvis treated with percutaneous resection.

## ARTICLE INFO

**Available at: http://www.intbrazjurol.com.br/video-section/20180490_Toussi_et_al**

**Int Braz J Urol. 2019; 45 (video #10): 640-640**

